# Prevalence and influencing factors of suicide in first-episode and drug-naive young major depressive disorder patients with impaired fasting glucose: a cross-sectional study

**DOI:** 10.3389/fpsyt.2023.1171814

**Published:** 2023-06-09

**Authors:** Yifan Li, Zhenjiang Liao, Qiuping Huang, Qianjin Wang, Honghong Ren, Xinxin Chen, Shuhong Lin, Chenhan Wang, Ying Tang, Jingyue Hao, Xuhao Wang, Hongxian Shen, Xiangyang Zhang

**Affiliations:** ^1^Department of Psychiatry, National Clinical Research Center for Mental Disorders, The Second Xiangya Hospital of Central South University, Changsha, Hunan, China; ^2^Department of Applied Psychology, School of Humanities and Management, Hunan University of Chinese Medicine, Changsha, China; ^3^The First Clinical Medical College of Lanzhou University, Lanzhou, China; ^4^CAS Key Laboratory of Mental Health, Institute of Psychology, Chinese Academy of Sciences, Beijing, China

**Keywords:** anxiety, depression disorder, suicide attempts, impaired fasting glucose, thyroid antibodies, blood lipids

## Abstract

**Background:**

An association exists between major depression disorder (MDD), suicide attempts, and glucose metabolism, but suicide attempts in young MDD patients with comorbid impaired fasting glucose (IFG) have been less well studied. The purpose of this study was to examine the prevalence and risk factors for suicide attempts in young, first-episode, drug-naive (FEDN) MDD patients with comorbid IFG.

**Methods:**

We recruited 917 young patients with FEDN MDD, 116 of whom were judged to have combined IFG because their blood glucose was >6.0. We collected anthropological and clinical data on all of them. The Hamilton Depression Scale (HAMD) score, the Hamilton Anxiety Scale (HAMA) score and the Positive and Negative Syndrome Scale (PANSS) positive subscale score were used to assess their clinical symptoms. Blood glucose, plasma thyroid function and lipid indicators were measured.

**Results:**

The prevalence of suicide attempts in young MDD patients with IFG was 32.8% (38/116). Furthermore, among young MDD patients with comorbid IFG, suicide attempters had more severe depression and anxiety symptoms, more comorbid psychotic symptom, higher levels of antibody of thyroid stimulating hormone and thyroid peroxidases (TPOAb), and more severe lipid metabolism disorders than those without suicide attempts. In addition, HAMA scores and TPOAb were independently associated with suicide attempts in young patients with FEDN MDD.

**Conclusion:**

Our study suggests that young MDD patients with IFG have a high rate of suicide attempts. Some clinical symptoms and thyroid function parameters may be the risk factor for suicide attempts in young MDD patients with impaired glucose metabolism.

## 1. Introduction

Major depressive disorder (MDD) is a common serious mental disorder in the world. MDD is characterized by persistent depressed mood, slowed thinking and reduced volitional activity, and in severe cases, even physical symptoms, psychotic symptoms and suicidal behavior (1). According to a recent systematic review, the lifetime prevalence of MDD ranges from 2 to 21% (2). According to the World Health Organization, as the second largest contributor to the global burden of disease, MDD causes enormous economic cost to individuals and society (3). MDD is not only a leading cause of disability globally, but is strongly associated with suicide attempts (4, 5). Suicide attempts are thought to be the most devastating outcome for people with depression, but the exact link between depression and suicide attempts remains unclear. Previous studies have linked several factors to suicide attempts in MDD patients, including young age, unemployment, psychotic symptoms, severe or persistent depressive symptoms, dyslipidemia, thyroid dysfunction, and glucose metabolism disorders (6–8). However, results have been inconsistent (9).

Multiple studies have shown a link between glucose metabolism and psychiatric disorders. For example, the prevalence of depression in people with diabetes generally ranges from 10 to 15%, approximately twice the prevalence of depression in people without diabetics (10). Individuals with MDD have also been found to have varying degrees of glucose metabolism disturbances, including elevated fasting blood glucose and changes in hormones that affect blood glucose, such as insulin, glucagon, adrenaline, and thyroid hormones (11, 12). One study found that patients with depression were 1.2–2.6 times more likely to develop diabetes than those without depression (13). Concerns have also been raised about the link between abnormal glucose metabolism and suicide attempts, which some studies suggest may reflect biological changes in individuals who have attempted suicide (14). Other studies have found that fasting glucose is higher in MDD patients with suicide attempts than in MDD patients without suicide attempts (15).

With the growing focus on depression, more research has been conducted on depressive symptoms and suicidal ideation in younger population than even before. According to a multinational study, the prevalence of MDD was estimated to range from 8.3 to 12.4% during the 12-month period between the ages of 18 and 33, while the overall prevalence of MDD during the 12-month period was only 6% (16, 17). A large population-based study in Singapore found that adults aged 18 to 34 years had a higher risk of MDD compared with other age groups (18). However, when it comes to metabolic disorders and suicide attempts, the most relevant studies have focused on the general population or older adults due to the low prevalence of metabolic disorders in young adults, ignoring the possible association between suicide attempts and abnormal glucose metabolism in younger MDD populations. Therefore, there is a need to focus on suicide attempts in young MDD patients with impaired fasting glucose (IFG).

In addition, suicide attempts may be influenced by MDD disease status and treatment, and may also show cross-cultural differences. For example, the suicide attempt rate for MDD in the United States is 36.3% compared to 20.1% in China (19–22). Therefore, recruiting asample consisting only of Han patients with first-episode and drug-naive (FEDN) MDD may provide a good opportunity to explore the incidence of suicide attempts in young MDD patients with IFG while minimizing the effects of confounding factors such as medication use and disease duration, with more reliable results (23). In this study, we intended to systematically analyze the clinical data and laboratory indicators of 18-35-year-old Han Chinese FEDN MDD patients with comorbid IFG, aiming to understand the incidence of suicide attempts and the relationship between suicide attempts and glycolipid metabolism, thyroid function, and mood disorders in this population. It is hoped that these results will help identify biomarkers of suicide attempts in young MDD patients with co-morbid IFG for early intervention.

## 2. Methods

### 2.1. Participants

After receiving approval from the Institutional Review Board of the First Hospital of Shanxi Medical University, we asked each participant or his or her guardian to carefully read and sign the informed consent form. From 2015 to 2017, patients were recruited from the psychiatric outpatient department of the First Hospital of Shanxi Medical University.

Inclusion criteria were: (1) age 18–35 years; (2) Han Chinese; (3) diagnosis of MDD, by two research psychiatrists through the Structure Clinical Interview for the Diagnostic and Statistical Manual of Mental Disorders, Fourth Edition (SCID); (4) no any previous medications; (5) at the first episode of MDD.

Exclusion criteria were: (1) conformed to a diagnosis of other psychiatric disorders on Axis I; (2) suffering from persistent infections, chronic diseases, immunosuppressive therapy, and other serious physical illnesses; (3) having substance use disorders, except for nicotine; and (4) pregnant and breastfeeding women.

### 2.2. Clinical interviews and assessments

Sociodemographic data, including age, sex, education, marital status, and duration of illness, were collected using a self-designed questionnaire.

The 17-item Hamilton Depression Scale (HAMD) was used to assess depressive symptoms, and a HAMD ≥24 points was considered to be a MDD patient (24). The 14-item Hamilton Anxiety Scale (HAMA) was used to assess anxiety symptoms and a HAMA ≥21 points was considered to have significant anxiety symptoms (25). The Positive and Negative Syndrome Scale (PANSS) positive subscale was used to assess psychotic symptoms, and a PANSS score ≥ 15 was considered to have psychotic symptoms (26). Two research psychiatrists were trained to assess the HAMD, HAMA, and PANSS prior to the study, and inter-rater correlation coefficients for HAMD, HAMA, and PANSS total scores were > 0.8.

Suicide attempts were defined as any potentially self-destructive behavior undertaken by the participants themselves that had some degree of threat of death but did not result in death (27–29). Two experienced psychiatrists assessed patients’ lifetime suicide attempts through face-to-face interviews, asking such a question: “Have you ever attempted suicide in your lifetime?” If the patient answered “yes,” he/she was considered as a suicide attempter. Then, the following details were collected: number of suicide attempts, date of each suicide attempt, and exact method.

### 2.3. Measurement of thyroid function and metabolic parameters

A blood sample was collected from each patient at 6:00 am to 8:00 am after an overnight fast. All samples were immediately sent to the hospital laboratory center to measure the following biomarkers: blood lipids including total cholesterol (TC), triglycerides (TG), high-density lipoprotein (HDL-C) and low-density lipoprotein (LDL-C), fasting blood glucose, and thyroid stimulating hormone (TSH), free triiodothyronine (FT3), free thyroxine (FT4), thyroid peroxidase antibody (TPOAb), and anti-thyroglobulin (TGAb).

World Health Organization (WHO) designated standards for diabetes and IFG were adopted due to their widespread use in China. IFG was defined as fasting glucose ≥6.1 mmol/L and < 7.0 mmol/L, whereas diabetes mellitus was defined as fasting glucose ≥7.0 mmol/L or postprandial glucose ≥11.1 mmol/L. Considering that diabetes is a worsening of impaired fasting glucose, in this study, IFG was defined as fasting glucose ≥6.1 mmol/L (30–32).

The suitable TG range was less than 1.76 mmol/L, 1.76–2.26 mmol/L was defined as slightly elevated, and above 2.27 mmol/L was defined as elevated. The suitable TC range was less than 5.18 mmol/L, 5.18–6.18 mmol/L was defined as mildly elevated and above 6.19 mmol/L was defined as elevated. The suitable LDL-C range was less than 3.37 mmol/L, 3.37–4.13 mmol/L was defined as mildly elevated, and more than 4.14 mmol/L was defined as elevated. The suitable HDL-C range was above 1.04 mmol/L, and below 1.04 mmol/L was defined as a decrease.

### 2.4. Statistical analysis

The Kolmogorov–Smirnov one-sample test was used to test whether the data conformed to a normal distribution. The exact odds ratio (OR) of suicide attempts in young MDD patients with and without IFG was calculated by multifactorial logistic regression, after excluding sociodemographic information. Young MDD patients with comorbid IFG were divided into two groups according to the presence or absence of suicide attempts. Independent samples t-test and chi-square test were used for quantitative and qualitative data, respectively, and Mann–Whitney test was used for non-normal or unequal variance data and stratified data. Pearson’s correlation coefficient was used to test the correlation between suicide attempts and clinical variables. Multifactorial logistic regression (Backward, Wald) was used to identify predictors of suicide attempts, and all variables related to suicide attempts in the bivariate correlation analysis were included as independent variables. Variance inflation factors (VIF) were used to determine multicollinearity between independent variables, with VIF > 5 indicating multicollinearity. SPSS version 26.0 (IBM, Chicago, IL, United States) was used for analysis. In this study, *p* < 0.05 (two-tailed) was considered statistically significant.

## 3. Results

### 3.1. Prevalence and details of suicide attempts in young MDD patients with comorbid IFG

We recruited a total of 917 patients with FEDN MDD under the age of 35 years, 116 of whom had a comorbid IFG. The rate of suicide attempts in the whole MDD patients was 19.5% (179/917), while the rate of suicide attempts in MDD patients with comorbid IFG was 32.8% (38/116). Furthermore, the rate of suicide attempts in MDD patients with IFG was 2.5 times higher than those without IFG (OR = 2.50, *p* < 0.001, 95% CI: 1.61–3.87).

We found that 92% of those who attempted suicide had committed suicide only once, and 8% had committed suicide twice. Among them, 41% attempted suicide by cutting their wrists, 34% attempted drug or gas overdose, 11% attempted suicide by jumping off a building, and 12% attempted suicide in traffic accidents. 76% of subjects attempted suicide within 2 weeks.

### 3.2. Sociodemographic data, clinical data, lipid metabolism, and thyroid function of suicide attempt and non-suicide attempt subgroups among MDD patients with comorbid IFG

Table 1 shows the differences in sociodemographic data, clinical data, lipid metabolism, and thyroid function between these two suicide attempt and non-suicide attempt subgroups. Of the 78 patients without suicide attempts, 55 (70.5%) were female, with a mean age of (24.21 ± 5.07) years, 38 (48.7%) were married, and the mean duration of illness was (4.77 ± 2.98) months. Of the 38 patients with suicide attempts, 25 (65.8%) were female, with a mean age of (24.53 ± 5.28) years, 16 (42.1%) were married, and the mean duration of illness was (4.28 ± 2.13) months. Although younger patients with MDD combined with IFG were more often male and single, older, had a shorter duration of disease, and lower education compared to those without suicide attempts, these differences were not statistically significant (*p* > 0.05).

**Table 1:** Socio-demographics and clinical characteristics between MDD comorbid IFG patients with or without suicide attempt.

Variable		MDD comorbid IFG		*t*/*χ*^2^/*Z*	*P*
		Without suicide attempt	With suicide attempt		
		*N* = 78	*N* = 38		
Sex	Male *n* (%)	23 (29.5%)	13 (34.2%)	0.27	0.61
	Female *n* (%)	55 (70.5%)	25 (65.8%)		
Marital status	Single *n* (%)	40 (51.3%)	22 (57.9%)	0.45	0.50
	Married *n* (%)	38 (48.7%)	16 (42.1%)		
Age [years, mean (SD)]		24.21 (5.07)	24.53 (5.28)	−0.32	0.75
Education	Elementary school and below *n* (%)	5 (6.4%)	6 (15.8%)	3.28	0.35
	Junior high school *n* (%)	37 (47.4%)	14 (36.8%)		
	Senior high school *n* (%)	30 (38.5%)	14 (36.8%)		
	University and above *n* (%)	6 (7.7%)	4 (10.5%)		
Duration of illness [months, mean (SD)]		4.77 (2.98)	4.28 (2.13)	0.91	0.36
Hamilton depression rating scale scores [mean (SD)]		30.78 (2.89)	32.42 (2.62)	−2.96	0.004
Hamilton anxiety rating scale scores [mean (SD)]		20.49 (3.02)	23.63 (3.22)	−5.15	<0.001
Psychotic symptoms	Without *n* (%)	72 (92.3%)	27 (71.1%)	9.23	0.002
	With *n* (%)	6 (7.7%)	11 (28.9%)		
Thyroid stimulating hormone [uIU/L, mean (SD)]		6.44 (2.66)	8.17 (2.59)	−3.33	0.001
Anti-thyroglobulinand [IU/L, mean (SD)]		98.95 (271.68)	183.95 (359.03)	−1.42	0.16
Thyroid peroxidases antibody [IU/L, mean (SD)]		66.34 (120.54)	215.82 (336.75)	−3.06	0.002
Free triiodothyronine [pmol/L, mean (SD)]		4.90 (0.71)	4.87 (0.66)	0.23	0.82
Free thyroxine [pmol/L, mean (SD)]		17.09 (2.90)	16.61 (3.17)	0.81	0.42
Fasting glucose [mmol/L, mean (SD)]		6.49 (0.42)	6.54 (0.38)	−0.69	0.49
Total cholesterol	Normal *n* (%)	27 (34.6%)	7 (18.4%)	2.50	0.01
	Marginally-elevated *n* (%)	25 (32.1%)	9 (23.7%)		
	Elevated *n* (%)	26 (33.3%)	22 (57.9%)		
Triglycerides	Normal *n* (%)	28 (35.9%)	9 (23.7%)	−1.43	0.15
	Marginally-elevated *n* (%)	15 (19.2%)	7 (18.4%)		
	Elevated *n* (%)	35 (44.9%)	22 (57.9%)		
Low density lipoprotein cholesterol	Normal *n* (%)	50 (64.1%)	21 (55.3%)	−1.35	0.18
	Marginally-elevated *n* (%)	18 (23.1%)	6 (15.8%)		
	Elevated *n* (%)	10 (12.8%)	11 (28.9%)		
High density lipoprotein cholesterol	Normal *n* (%)	48 (61.5%)	12 (31.6%)	9.19	0.002
	Decreased *n* (%)	30 (38.5%)	26 (68.4%)		

Among young MDD patients with IFG, suicide attempters had higher HAMD scores (*t* = −2.96, *p* = 0.004), HAMA scores (*t* = −5.15, *p* < 0.001) and psychotic symptoms (*χ*2 = 9.23, *p* = 0.002) more TSH (*t* = −3.33, *p* = 0.001), as well as higher levels of TPOAb (*χ*2 = −3.06, *p* = 0.002), TC (*Z* = 2.50, *p* = 0.01), but lower HDL-C levels (*χ*2 = 9.19, *p* = 0.002) than non-suicide attempters.

### 3.3. Identification of risk factors for suicide attempts in young MDD patients with IFG

**Figure 1:**
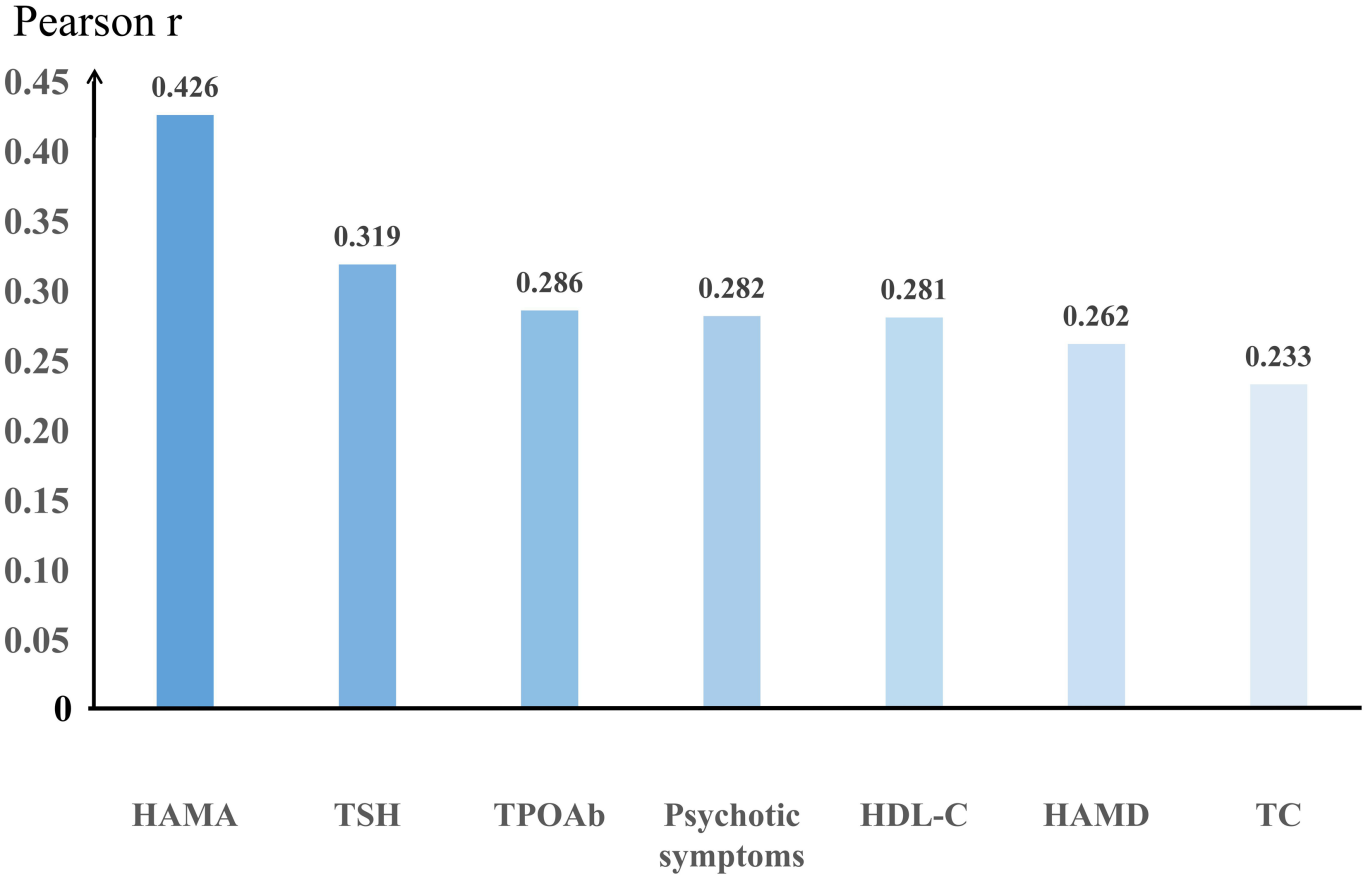
Results of bivariate correlation analysis significantly associated with suicide attempts.

Bivariate correlation analysis showed that suicide attempts were significantly associated with the following variables: HAMD scores (*r* = 0.267; *p* = 0.004), HAMA scores (*r* = 0.434; *p* < 0.001), psychotic symptoms (*r* = 0.282; *p* = 0.002), TSH (*r* = 0.297; *p* = 0.001), TPOAb (*r* = 0.311; *p* = 0.001), TC (*r* = 0.230; *p* = 0.013), and HDL-C (*r* = 0.281; *p* = 0.002), see Figure 1.

Further, we performed multivariate logistic regression (Back, Wald) to examine risk factors for suicide attempts, all variables were included as independent variables. Table 2 shows that the independent risk factors for suicide attempts in young MDD patients with IFG were: HAMA scores (OR = 1.377, *p* < 0.01, 95% CI = 1.171–1.620) and TPOAb (OR = 1.003, *p* = 0.013, 95% CI = 1.001–1.005). The VIF for all results was less than 5, indicating that the problem of covariance between variables was not severe.

**Table 2:** Predictors of suicide attempt in major depression disorder comorbid impaired fasting glucose patients.

			Coefficients			95% Confidence interval for OR	
	*β*	Std. error	Wald	*P*	OR	Lower bound	Higher bound
Hamilton anxiety rating scale scores	0.320	0.083	14.898	<0.001	1.377	1.171	1.620
Thyroid peroxidases antibody	0.003	0.001	6.201	0.013	1.003	1.001	1.005
High density lipoprotein cholesterol	0.834	0.481	3.015	0.082	2.304	0.898	5.908

## 4. Discussion

To our knowledge, this is the first study to examine the incidence of suicide attempts and associated factors in young FEDN MDD patients with comorbid IFG. The main findings of this study were as follows: (1) The incidence of suicide attempts was 19.5% (179/917) in young FEDN MDD patients, while the incidence of suicide attempts in young MDD patients with IFG was 32.8% (38/116), which was significantly higher than in young MDD patients without IFG. (2) Young MDD patients with IFG who attempted suicide had higher HAMD and HAMA scores, more psychotic symptoms, higher TSH, TPOAb, and TC levels, but lower HDL-C levels. (3) HAMA scores and TPOAb were the risk factor for suicide attempts in young MDD patients with IFG.

In the present study, the rate of suicide attempts in young FEDN MDD patients with IFG was 32.8%. Previous studies showed that the suicide attempt rate among depressed patients in China ranged from 18.5 to 23.5% (33, 34). In Korea and Thailand, 19.8 and 16.9% of depressed patients reported suicide attempt, respectively (35, 36), while in France, 33.7% of patients with depression or bipolar disorder reported lifetime suicide attempts (37). Our study showed that the rate of suicide attempts among young Chinese MDD patients is much higher than that in the general Asian depression population. We propose that there are several reasons for the different prevalence rates of suicide attempts. First, IFG or diabetes may have a direct or indirect effect on the occurrence and development of suicide attempts (14). Second, the inconsistency of age among patients will also have an impact. For MDD people aged 35 and under, these younger subjects may be more impulsive and more likely to have thoughts of ending their lives (38). Third, some of the previous studies recruited inpatients, a population in which suicide attempts may change after receiving certain treatments, and we recruited MDD outpatients rather than inpatients, which also introduced differences in outcomes. Fourth, different social and cultural backgrounds may also have an impact. Because of traditional influences, Asians are more introverted than Europeans or Americans, and they are less likely to express their emotions, which is a possible reason for the difference. Fifth, different ethnic groups have different gene frequencies of suicide-related gene polymorphisms, and different economic and medical conditions may also have different identification and interventions for suicide attempts (39, 40).

Glucose metabolic function is often closely related to emotional state, and our study showed that MDD patients with IFG were more likely to have some means of ending their lives. Several studies have found that patients with diabetes have a higher risk of suicide than normal controls (41). These studies consistently reflect an intrinsic link between impaired blood glucose and suicide attempts. One possible explanation is that depressed patients with suicidal thoughts are more prone to substance abuse and lifestyle disorders that lead to impaired glucose and lipid metabolism. Our previous study showed that among MDD patients with psychotic symptoms, those who attempted suicide had higher glucose levels than those who did not, and this study reached similar conclusions in young adults (42). Therefore, there is a need for increased attention and prevention of suicide attempts in MDD populations with comorbid IFG in order to avoid serious consequences.

Suicide is the most serious consequence of depression and is considered one of the criteria to assess the severity of depression (43). In our study, HAMD scores were significantly higher in young MDD combined with IFG patients with suicidal attempts than in patients without suicidal attempts in young MDD combined with IFG patients. Several studies have confirmed that the risk of suicide attempts increases with increasing levels of depression. For example, Berardis et al. found that suicide attempts were positively associated with HAMD in adults with first-episode MDD (44). Furthermore, Hoertel et al. conducted a 3-year cohort study and found that severity of depression was an independent risk factor for suicide attempts in MDD patients (45). Our findings are consistent with previous studies and reflect that the HAMD as a scale can be a good indicator of suicide-related behaviors and help determine the severity of depression in young MDD patients and even in other MDD populations.

This study found that patients who attempted suicide had higher levels of anxiety symptoms, and it was also an independent risk factor for suicide attempts in young MDD patients with IFG. In fact, research on whether anxiety increases the risk of suicide in MDD patients is inconsistent. Several studies have reported a strong association between anxiety and suicide attempts. For example, Batterham et al. found that anxiety symptoms were associated with a greater overall risk of suicide attempt in depressed patients in a large longitudinal cohort study (46). Goldberg et al. and Pfeiffer et al. also found consistent findings in samples from different countries (47, 48). Furthermore, a retrospective study reported that 79% of patients had severe anxiety or agitation prior to suicide (49). However, some studies have reported inconsistent results. For example, Abreu et al. (50) ound that comorbid anxiety symptoms were not associated with suicide attempts in MDD patients. Furthermore, Xin et al. found that MDD patients without anxiety symptoms had higher rates of suicide attempts than MDD patients with anxiety symptoms, and Placidi even found that anxiety symptoms were a protective factor for suicide attempts in MDD patients (51, 52). All these differences may be due to the following reasons. First, our study recruited people aged 35 years and younger, who may have higher impulsivity and even extreme behavior (38). Second, these studies came from different countries with differences due to sociocultural factors, such as the higher acceptance of suicide in Japan than in Western Christian countries (53). Third, in this study, we recruited all patients with FEDN MDD, and many studies have samples from hospitalized patients, where outcomes may change after medication (54). Therefore, more follow-up studies should focus on the underlying links between anxiety and suicide attempts.

In the present study, we found that MDD patients with IFG were more likely to experience psychotic symptoms during suicide attempts. Our findings are consistent with previous studies. For example, Ma et al. found a significant association between suicide attempts and psychotic symptoms (21), while Sachs-Ericsson et al. also found consistent results in older patients with depression (55). Furthermore, our findings are supported by systematic reviews and meta-analyses (56). We hypothesize that one of the possible links between suicide attempts and psychotic symptoms is avoidance. Suicide attempts can be seen as a way for people with MDD to avoid psychotic symptoms (57). However, some studies have found no association between psychotic symptoms and suicide attempts (58). One explanation for this discrepancy is the clinical heterogeneity of the sample, such as outpatients versus inpatients and different stages of illness (56, 59). Notably, this study found that psychotic symptoms were not an independent risk factor for suicide attempts, whereas anxiety and depression were. We hypothesize that, as previously mentioned, MDD patients with psychotic symptoms experience greater distress, severe anxiety, and depression, and that these emotional problems are more likely to motivate MDD patients with psychotic symptoms to take extreme suicidal measures.

The link between mood disorders and changes in the hypothalamic–pituitary-thyroid (H4PT) axis has been recognized, and this study found that suicidal individuals had higher TSH, a hormone secreted by the pituitary gland that is primarily responsible for regulating thyroid cell proliferation, thyroid blood supply, and thyroid hormone synthesis and secretion. However, previous studies have shown inconsistent results regarding TSH and suicide attempts in patients with MDD. It was found that higher plasma TSH and cortisol levels in depressed patients may trigger suicide attempts (60). Our earlier study also found an independent association between TSH and suicide attempts in FEDN MDD patients with anxiety symptoms (23). However, suicide attempts in MDD patients were found to significantly reduce serum TSH levels (61). The contradictory results may be due to changes in thyroid function in patients with IFG or diabetes, with studies reporting that subclinical hypothyroidism worsens glycemic control in diabetic patients, who have a higher prevalence of thyroid dysfunction, especially in patients with type 1 diabetes (62). In addition, differences in age distribution and sample size can also cause confounding. Finally, different collection times will introduce differences in TSH due to biological rhythms.

Several studies have been conducted on antithyroid antibodies in the MDD patients, but none of them have addressed TPOAb, IFG and suicide attempts in MDD patients. We found that TPOAb was significantly higher in young FEDN MDD patients with than without IFG, and remained an independent risk factor for suicide attempts after accounting for other factors. TPOAb, an autoantibody to the thyroid, when elevated, indicates an increased risk of autoimmune thyroid disease. We sought to explain the association between TPOAb and suicide attempts in MDD patients with IFG. First, MDD patients with IFG in combination with suicide attempts may also have autoimmune thyroid diseases such as Graves’ disease and Hashimoto’s thyroiditis, and MDD patients with these diseases have increased rates of suicide attempts in addition to glycemic instability (63). A recent large cohort study found increased rates of suicide in patients with Graves’ disease (64), and another large study of patients with thyroiditis also found increased rates of suicide (65). An Another possible reason is that anxiety or depression acts as a mediator between TPOAb and suicide, which previous studies have reported to be associated with an increased risk of suicide (66, 67). However, frankly, the βof TPOAb in the regression model was very close to 1, suggesting that the association between TPOAb and suicide may also be brought about by bias in the study, and therefore the association between TPOAb and suicide should be explored in more appropriate studies.

In our study, suicide attempts were found to be associated with elevated TC and lower HDL in MDD patients with IFG, suggesting an increased risk of hyperlipidemia in these individuals. Previous studies have also found an association between hyperlipidemia and suicide attempts in MDD patients. For example, A large sample of middle-aged Japanese men found that TC was associated with the risk of long-term suicide attempts (68), while a recent study found that suicide attempts were associated with high TC and low HDL levels in MDD patients (69). A hypothesis regarding polyunsaturated fatty acids (PUFA) may explain the association between hyperlipidemia and suicide attempts. Omega-3 (n-3) and omega-6 (n-6) PUFAs have important functions in synapse formation, neurotransmission and signal transduction in the central nervous system (70). It has been found that an increase in the n-6 PUFA/n-3 PUFA ratio may lead to altered 5-hydroxytryptamine (5-HT) transporter binding and dysregulation of the hypothalamic–pituitary–adrenal axis (HPA axis) (71, 72), the latter two of which have been shown to be associated with suicide in many studies (28, 73). We therefore hypothesize that for the hyperlipidaemic MDD population, as TC is elevated and HDL is decreased, and as n-3 PUFA is negatively correlated with the serum TC/HDL ratio, then n-3 PUFA decreases and the n-6/n-3 ratio increases, indirectly elevating the risk of suicide. However, studies have also found an association between lower TC and suicide (74). When cholesterol falls, cell membrane fluidity decreases and 5-HT receptors fall, leading to increased impulsivity and suicidal behavior (75, 76). Thus, the lipid-suicide relationship may be complex and there are important mediators, such as 5-HT, and the results of this study should be considered preliminary, with follow-up large-scale studies exploring the association between 5-HT, the HPA axis, abnormal lipids and suicide attempts.

The current study has some obvious limitations. First, considering that the WHO system criteria for diabetes and IFG are widely used in China, these criteria were used in the present study, however, the criteria for diabetes and IFG developed by the American Diabetes Association (ADA) are also widely used (77), so we carefully analyzed the entire statistics according to the ADA criteria (specific details are in the supplemental file) and some few but important ones do exist between the two systems. Therefore, when quoting these results, careful confirmation of which diagnostic system’s criteria were used is needed. Second, this study is a cross-sectional study. Although factors associated with suicide attempts in young MDD patients with comorbid IFPG were identified, causality was difficult to determine and, in addition, the lack of longitudinal data led to the inclusion of patients with bipolar disorder without a history of manic episodes as a possible risk. Future longitudinal studies should be conducted in young MDD to explore risk factors for suicide and exclude patients with bipolar disorder. Third, considering (1) the strong association between 5-HT and suicide and (2) the importance of the context and causes at the time of suicide attempts, the lack of relevant indicators in this study limits the exploration of suicide mechanisms in young people with comorbid IFG. Follow-up studies should include these indicators in order to gain a deeper understanding of suicide in these individuals. Fourth, the present study excluded substance use disorders, severe personality disorders, and pregnant women because these three groups may be more vulnerable in the face of depression, and thus their exclusion may produce biased outcomes. Fifth, due to the low prevalence of metabolic disorders in younger populations, only 116 MDD patients with IFD were recruited in this study, and only 12 of these subjects had fasting glucose >7.0. A multicenter study with a larger sample could have been conducted to obtain more reliable results, especially the association between diabetes and suicide.

## 5. Conclusion

Overall, this study showed that the rate of suicide attempts in young MDD patients with comorbid IFG (32.8%) was significantly higher than that in those without IFG. MDD patients with suicide attempts had more severe depressive symptoms, a higher rate of comorbid anxiety symptoms or psychotic symptoms, and a higher rate of thyroid dysfunction and hyperlipidemia than those without suicide attempts. HAMA scores and TPOAb were independently associated with suicide attempts in young MDD patients with IFG. However, due to the relatively small sample size and limitations of the cross-sectional design, the results should be interpreted with caution, and future confirmation of the results using a longitudinal design in a larger sample size is needed.

## Data availability statement

The original contributions presented in the study are included in the article/Supplementary material, further inquiries can be directed to the corresponding authors.

## Ethics statement

The research protocol was approved by the Institutional Review Board of the First Clinical Medical College of Shanxi Medical University (No. 2016-Y27). All the participants understood the purpose and procedure of the study and signed the informed consent form. The patients/participants provided their written informed consent to participate in this study.

## Author contributions

XZ and HS designed and supervised the study. QW, HR, ZL, XC, SL, YT, JH, and XW collected the data. CW analyzed and interpreted the data. YL drafted the manuscript. QW and QH revised the manuscript. XZ was responsible for the critical revision of the manuscript. All authors contributed to the article and approved the submitted version.

## Funding

This work was supported by the National Natural Science Foundation of China (No. 81971249), and National Key R&D Program of China (No. 2020YFC2005300).

## Conflict of interest

The authors declare that the research was conducted in the absence of any commercial or financial relationships that could be construed as a potential conflict of interest.

## Publisher’s note

All claims expressed in this article are solely those of the authors and do not necessarily represent those of their affiliated organizations, or those of the publisher, the editors and the reviewers. Any product that may be evaluated in this article, or claim that may be made by its manufacturer, is not guaranteed or endorsed by the publisher.
