# Supplementary material for: Prevalence and influencing factors of suicide in first-episode and drug-naive young major depressive disorder patients with impaired fasting glucose: a cross-sectional study
Source: Front Psychiatry. 2023 Jun 9;14:1171814. doi: 10.3389/fpsyt.2023.1171814 (PMC10289199; doi:10.3389/fpsyt.2023.1171814)
Supplement: Supplementary file 1 [file Data_Sheet_1.pdf]

Supplementary file

We have re-analysed our statistics according to the criteria for impaired fasting glucose (5.6 mmol/L-6.9 mmol/L) set by the American Diabetes Association. After re-analysing the results, we found that when the criteria for IFG were changed, young depressed patients with IFG who had suicidal attempts showed new group differences in fasting glucose from those depressed without suicidal attempts, and other variables that differed were consistent with those previously; in addition, we found that when the criteria for IFG were changed, in the young first-episode and drug-naive MDD population with IFG, HAMA scores, TPOAb and TSH could independently identify suicide attempts. We speculate that the difference between the before and after results may stem from the heterogeneity of the IFG patients included due to the different IFG criteria. For details, please see below.

S 1.0 Prevalence and details of suicide attempts in young MDD patients with comorbid IFG

We recruited a total of 917 patients with FEDN MDD under the age of 35 years, 315 of whom had a comorbid IFG. The rate of suicide attempts in the whole MDD patients was 34.4% (315/917), while the rate of suicide attempts in MDD patients with comorbid IFG was 27.6% (87/315). Furthermore, the rate of suicide attempts in MDD patients with IFG was 2.08 times higher than those without IFG (OR=2.08, P<0.001, 95% CI: 1.48-2.91).

We found that 74% of those who attempted suicide had committed suicide only once, 16% had committed suicide twice and three times. Among them, 38% attempted suicide by cutting their wrists, 39% attempted drug or gas overdose, 10% attempted suicide by jumping off a building, and 14% attempted suicide by traffic accidents or other ways. 56% of subjects attempted suicide within two weeks.

S 2.0 Sociodemographic data, clinical data, lipid metabolism and thyroid function of suicide attempt and non-suicide attempt subgroups among MDD patients with comorbid IFG

S.Table 1 shows the differences in sociodemographic data, clinical data, lipid metabolism and thyroid function between these two suicide attempt and non-suicide attempt subgroups. Of the 228 patients without suicide attempts, 147 (64.5%) were female, with a mean age of (24.66 ± 5.44) years, 109 (47.8%) were married, and the mean duration of illness was (5.08 ± 3.26) months. Of the 87 patients with suicide attempts, 55 (63.2%) were female, with a mean age of (24.83 ± 5.17) years, 37 (42.5%) were married, and the mean duration of illness was (5.45 ± 4.28) months. Age, sex, marital status, education and duration of illness did not differ significantly between the two groups.

Among young MDD patients with IFG, suicide attempters had higher HAMD

scores (t=6.00, P=0.004), HAMA scores (t=8.32, P<0.001) and fasting glucose (t = -2.38 , P = 0.02), more psychotic symptoms (χ^2 = 17.18, P<0.001), TSH (t = -7.18, P<0.001), as well as higher levels of TPOAb (Z=5.55, P<0.001), TC (Z = -4.04, P<0.01), but lower HDL-C levels (χ^2 = 8.12, P = 0.004) than non-suicide attempters.

S 3.0 Identification of risk factors for suicide attempts in young MDD patients with IFG

Bivariate correlation analysis showed that suicide attempts were significantly associated with the following variables: HAMD scores (r=0.309; P<0.001), HAMA scores (r=0.403; P<0.001), psychotic symptoms (r=0.234; P<0.001), TSH (r=0.376; P<0.001), fasting glucose (r=0.166, P=0.003), TGAb (r=0.281, P<0.001), TPOAb (r=0.266; P<0.001), TC (r=0.228; P<0.001), and HDL-C (r=0.161; P=0.004).

Further, we performed multivariate logistic regression (Back, Wald) to examine risk factors for suicide attempts, all variables were included as independent variables. S.Table 2 shows that the independent risk factors for suicide attempts in young MDD patients with IFG were: HAMA scores (OR=1.299, P<0.001, 95% CI=1.171-1.434), TPOAb (OR=1.004, P<0.001, 95% CI = 1.002-1.006), and TSH (OR=1.254, P=0.001, 95% CI=1.093-1.439). The VIF for all results was less than 5, indicating that the problem of covariance between variables was not severe.

S.Table 1 Socio-demographics and clinical characteristics between MDD comorbid IFG patients with or without suicide attempt

Variable		MDD comorbid IFG		$t/\chi^2/Z$	P
		Without suicide attempt N=228	With suicide attempt N=87		
Sex	Male n(%)	81 (35.5%)	32 (36.8%)	0.43	0.84
	Female n(%)	147 (64.5%)	55 (63.2%)		
Marital status	Single n(%)	119 (52.2%)	50 (57.5%)	0.71	0.40
	Married n(%)	109 (47.8%)	37 (42.5%)		
Age (years,mean[SD])		24.66 (5.44)	24.83 (5.17)	-0.25	0.80
Education	Elementary school and below n(%)	10 (4.4%)	9 (10.3%)	-0.64	0.52
	Junior high school n(%)	121 (53.1%)	36 (41.4%)		
	Senior high school n(%)	82 (36.0%)	31 (35.6%)		
	University and above n(%)	15 (6.6%)	11 (12.6%)		
Duration of illness (months,mean[SD])		5.08 (3.26)	5.45 (3.52)	-0.90	0.37

Hamilton Depression Rating Scale (mean[SD])		30.57 (2.68)	32.59 (2.62)	-6.00	<0.001
Hamilton Anxiety Rating Scale (mean[SD])		20.29 (3.01)	23.64 (3.66)	-8.32	<0.001
Psychotic symptoms	Without n(%)	210 (92.1%)	65 (74.7%)	17.18	<0.001
	With n(%)	18 (7.9%)	22 (25.3%)		
Thyroid stimulating hormone (uIU/L,mean[SD])		5.53 (2.27)	7.66 (2.55)	-7.18	<0.001
Anti-thyroglobulinand (IU/L,mean[SD])		90.60 (239.70)	153.32 (290.18)	-1.96	0.05
Thyroid peroxidases antibody (IU/L,mean[SD])		55.48 (108.59)	182.67 (298.88)	-5.55	<0.001
Free triiodothyronine (pmol/L,mean[SD])		4.97 (0.72)	4.88 (0.79)	0.93	0.36
Free thyroxine (pmol/L,mean[SD])		16.80 (3.01)	16.69 (3.36)	0.27	0.79
Fasting glucose (mmol/L,mean[SD])		6.03 (0.42)	6.17 (0.43)	-2.38	0.02
Total cholesterol	Normal n(%)	89 (39.0%)	19 (21.8%)	-4.04	<0.001
	Marginally-elevated n(%)	86 (37.7%)	27 (31.0%)		
	Elevated n(%)	53 (23.2%)	41 (47.1%)		
Triglycerides	Normal n(%)	98 (43.0%)	26 (29.9%)	-1.91	0.06
	Marginally-elevated n(%)	42 (18.4%)	20 (23.0%)		
	Elevated n(%)	88 (38.6%)	41 (47.1%)		
Low density lipoprotein cholesterol	Normal n(%)	146 (64.0%)	48 (55.2%)	-1.68	0.09
	Marginally-elevated n(%)	53 (23.2%)	21 (24.1%)		
	Elevated n(%)	29 (12.7%)	18 (20.7%)		
High density lipoprotein cholesterol	Normal n(%)	150 (65.8%)	42 (48.3%)	8.12	0.004

	Decreased n(%)	78 (34.2%)	45 (51.7%)

S.Table 2 Predictors of suicide attempt in major depression disorder comorbid impaired fasting glucose patients

			Coefficients			95% Confidence Interval for OR	
	β	std. error	wald	P	OR	Lower bound	Higher bound
Hamilton AnxietyRating Scale	0.262	0.050	27.259	<0.001	1.299	1.178	1.434
Thyroid peroxidases antibody	0.004	0.001	14.461	<0.001	1.004	1.002	1.006
Thyroid stimulating hormone	0.226	0.070	10.411	0.001	1.254	1.093	1.439
Anti-thyroglobulinand	-0.001	0.001	3.351	0.067	0.999	0.997	1.000
